# Effect of Heat Treatment on Corrosion Resistance of A356 Casting Alloy

**DOI:** 10.3390/ma18051056

**Published:** 2025-02-27

**Authors:** Kyung-Su Jang, Taehwan Jang, Hyunbin Jo, Dongmin Shin, Soomin Lee, Sung-Dae Kim, Se-Hun Kwon, Junghoon Lee

**Affiliations:** 1 School of Materials Science & Engineering, Pusan National University, Busan 46241, Republic of Korea; dc2000x@hanmail.net; 2 Department of Metallurgical Engineering, Pukyong National University, Busan 48513, Republic of Korea; taehwan77@pknu.ac.kr (T.J.); jhbin@pukyong.ac.kr (H.J.); shin2202@pukyong.ac.kr (D.S.); smlee7@pukyong.ac.kr (S.L.); 3 Department Materials Science and Engineering, Pukyong National University, Busan 48513, Republic of Korea; sdkim@pknu.ac.kr

**Keywords:** A356 alloy, solution treatment, aging, corrosion, hardness

## Abstract

Al-Si alloy, known for its excellent corrosion resistance and high strength, is utilized across various industries, such as electric vehicles, requiring light metal parts for efficiency. Heat treatment is an effective process for enhancing the performance of Al-Si alloys by controlling the microstructure and precipitation. Optimized heat treatment improves the mechanical properties of Al-Si alloy, including strength, hardness, and corrosion resistance. In this study, the effect of heat treatment on the microstructure, corrosion resistance, and hardness of A356 casting alloy, which has recently been used in electric vehicles, was analyzed. The as-cast A356 alloy was used as a control sample, with a solid solution treatment, followed by aging treatment. The results showed that the hardness and corrosion resistance of A356 alloy can be changed by controlling the formation and distribution of Mg_2_Si precipitates within the microstructure through the solution heat treatment and following aging. Additionally, the distribution and strengthening mechanism of Mg_2_Si precipitates were examined using XRD. With increasing aging time, more Mg_2_Si precipitates were formed, increasing hardness. However, the precipitation of Mg_2_Si reduces the corrosion resistance of A356 alloy. Thus, it was revealed that the heat treatment of A356 alloy for enhancing mechanical properties by precipitating Mg_2_Si causes a side effect of corrosion resistance.

## 1. Introduction

Aluminum alloys, with a high strength-to-weight ratio, excellent corrosion resistance, and ease of recycling, have been extensively studied and applied as essential materials that can enhance energy efficiency in modern industries [1,2,3]. Aluminum casting alloys are widely used in the production of major structural components, including automobile wheels, suspension parts, and engine mounts, due to their excellent castability and cost-effectiveness. In particular, due to their lightweight and specific strength, aluminum casting alloys are used in various casting parts in the aerospace and electronics industries [4,5,6,7]. In particular, the rapid growth of the electric vehicle (EV) market has underscored the significance of Al-Si casting alloys, as reducing vehicle weight and enhancing fuel efficiency have become critical concerns [8,9,10].

Due to this extensive demand in industrial fields, research on aluminum casting alloys has been widely conducted to improve the mechanical properties, corrosion resistance, productivity, castability, surface finishing, and recycling. Moreover, although aluminum alloys are advantageous for reducing vehicle weight, research is primarily focused on increasing the strength of casting alloys, as enhancing their strength would enable further weight reduction. Ongoing research aims to improve the performance of existing cast products by incorporating modifying elements, such as Nd, Sc, and TiC, to enhance corrosion resistance [11,12,13]. Additionally, earth’s rare elements, such as La, Pr, Nd, Sm, Gd, Tb, Dy, Er, and Ce, along with transition metals, such as Zr, Mn, Cr, and Ag, improve mechanical properties [14,15,16,17]. This approach offers a range of alloy design options and enhances the physical and mechanical properties of the material by influencing its microstructure and precipitate formation.

Among various Al-Si casting alloys, A356 alloy is used in electric vehicles for battery-related applications such as frames, batter casing, and cover; thus, extensive research has been conducted on the A356 alloy. Due to the contents of Si and Mg, A356 alloy has superior castability as well as mechanical properties. Additionally, the mechanical properties of A356 alloy can be optimized by controlling the microstructure through heat treatment techniques, including solution treatment, aging, or a combination of both. The mechanical properties of A356 alloy are greatly affected by its microstructure, and, in particular, fatigue resistance, tensile strength, and ductility are limited by the dendritic structure and nonuniformly distributed plate-like Si particles [18]. The cast A356 alloy has limitations in mechanical performance due to the formation of primary alpha (α-Al) dendrites and acicular eutectic Si, which limits its industrial applications. To solve these problems and improve performance, microstructure control is essential, and various heat treatment processes are applied to overcome these defects [19]. Heat treatment is a crucial process that effectively controls microstructure and enhances properties such as strength, hardness, and corrosion resistance without the addition of new elements. It is increasingly being utilized as a practical method to optimize material performance while simplifying alloy design. In particular, heat treatment technology has been studied as a straightforward yet effective method for enhancing performance. This process can improve mechanical properties by optimizing grain size and internal stress through the control of precipitation and distribution in a supersaturated solid solution. The microstructure and mechanical properties of A356 alloy vary significantly based on the heat treatment conditions. In particular, the T6 heat treatment, which involves a solution treatment followed by aging, can influence material properties such as strength and ductility by controlling the precipitated phases, such as Mg_2_Si, within the microstructure [20,21,22]. However, the effect of heat treatment on corrosion resistance, which is critical for aluminum alloys, has been rarely reported for A356 alloys.

In this study, the change in the corrosion resistance of A356 alloy with respect to the heat treatment was investigated. The effects of various heat treatment conditions on the microstructure and hardness of A356 alloy were analyzed. In particular, the effects of Mg_2_Si precipitation, which significantly affects the mechanical properties of A356 alloy, were explored. To achieve this, the microstructural changes in A356 alloy through solution and aging treatments from the as-casted state are compared. The distribution of precipitates, such as Mg_2_Si, and the associated strengthening mechanisms were examined using X-ray diffraction (XRD) analysis. The relationship between hardness and corrosion resistance was confirmed and optimized through the formation of precipitates in the microstructure as a result of heat treatment. Transmission electron microscopy (TEM) analysis was also performed to confirm the presence of Mg_2_Si. Reverse potential polarization and electrochemical impedance spectroscopy (EIS) were performed to investigate the effect of heat treatment on corrosion resistance. The results and discussion on the microstructure and corrosion resistance would contribute to the design of heat treatment of A356 casting alloy for better performance in various applications.

## 2. Materials and Methods

In this study, the specimens were prepared by pouring molten aluminum alloy, melted at 687 °C, into a pre-designed mold and processing it through a die casting procedure, which is generally used in manufacturing of A356 alloy parts. The mold was preheated to 400 °C to minimize casting defects and induce uniform solidification. Through the analysis results using an Optical Emission Spectrometer, it was confirmed that A356 with the chemical composition ratio shown in Table 1 was produced. The A356 specimen was cut into dimensions of 15 mm × 15 mm × 5 mm.

Before each heat treatment process, JMatPro simulation using the chemical composition in Table 1 was utilized to predict the phase changes of the A356 alloy to find background of solution and aging treatment conditions. The solution treatment was conducted at 525 °C, and the aging treatment was performed at 190 °C. Detailed conditions of heat treatments are summarized in Table 2. Vickers’ hardness tester was used to measure the hardness.

Potentiodynamic polarization experiments were conducted using a three-electrode electrochemical cell and a VersaStat 3 potentiostat (AMETEK Scientific Instruments, Oak Ridge, TN, USA). Ag/AgCl electrode saturated in KCl (E = 0.197 V) was employed as the reference electrode, and Pt electrode was utilized as the counter electrode. Further, 3.5 wt.% NaCl (99%, Duksan Pure Chemicals, Ansan, Republic of Korea) solution was employed for corrosive electrolyte. The experiment began by measuring the Corrosion Open-Circuit Potential (OCP) for 600 s to stabilize the corrosion potential. This step was performed to confirm the electrochemical stability state on the sample surface and to minimize initial potential fluctuation. The potentiodynamic polarization measurements were conducted from an initial potential of −1.0 V to a final potential of −0.2 V, and the time per point was set to 1 s to ensure high time reliability of the data.

Before the electrochemical measurements, each specimen was polished up to 2000 grit with SiC sandpaper. The corrosion resistance of the specimen and its electrochemical behavior at the interface were evaluated through electrochemical impedance spectroscopy (EIS) analysis. EIS analysis was performed using a three-electrode cell configuration. Ag/AgCl was used as the reference electrode, Pt as the counter electrode, and the working electrode was set for each specimen. For system stability during EIS measurement, the Open-Circuit Potential (OCP) was monitored for 20 min, and the data were set to 1 point per second. In the potentiostatic EIS mode, measurements were performed in a frequency range from 100,000 Hz to 0.1 Hz with 10 mV amplitude, and the data quality was set to the reference value 3 to secure high resolution and reliability. To minimize the experimental error in electrochemical corrosion analysis, seven samples fabricated in each condition were tested. Excluding potentiodynamic polarization curves with maximum and minimum corrosion current density for each condition, five corrosion current densities were averaged. In case of EIS, after model fitting of seven results, an averaged charge transfer resistance was estimated and summarized, excluding the maximum and minimum value. Then, potentiodynamic polarization and EIS curves, which have the most similar values (i.e., corrosion current density and charge transfer resistance) to average, were shown as representative results.

## 3. Results and Discussion

### 3.1. Microstructural Analysis Following Heat Treatment of A356 Alloy

**Figure 1:**
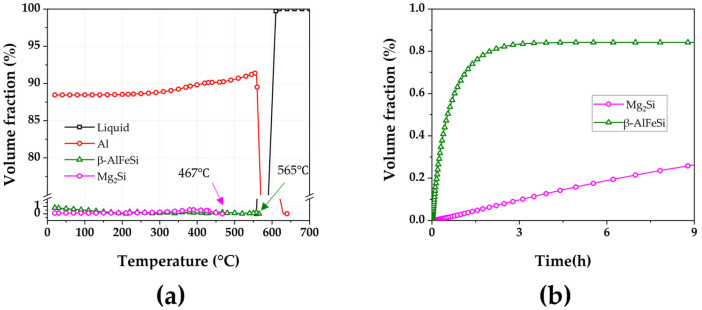
Calculation and prediction of precipitate phases in A356 alloy based on heat treatment process using JmatPro6 Software (version 9): (**a**) volume fraction of phase with respect to the temperature and (**b**) volution fraction of phase at 190 °C with respect to the duration time.

Figure 1 shows the results of JMatPro simulation based on the chemical composition of the A356 alloy (Table 1). During the die casting process, various intermetallic compounds can be precipitated, especially during the cooling process of the solidified metal, and these precipitated phases can affect the mechanical and corrosion performance. Therefore, solution treatment is essential to control the precipitated phase. The Mg_2_Si phase is not present over a temperature of 467 °C (Figure 1a), indicating that the Mg_2_Si phase can be dissolved into the matrix over that temperature in the equilibrium phase diagram. Therefore, the temperature of solution treatment (525 °C) in this study would effectively make the as-casted alloy a solid solution. In addition, the β-AlFeSi phase would not be dissolved into the matrix before the materials melted at 565 °C, and the volume fraction is almost constant over the temperature. Therefore, the fraction of the β-AlFeSi phase would not be controlled by a post-heat treatment. Assuming the A356 alloy is a totally solid solution, the volume fraction of the Mg_2_Si phase increases with time at 190 °C. In addition, the aging of Al-7Si-0.3Mg alloy was conducted at 160–200 °C for 4–15 h [19,21,23]. Therefore, the aging conditions in Table 2 can effectively promote the precipitation of the Mg_2_Si phase.

**Figure 2:**
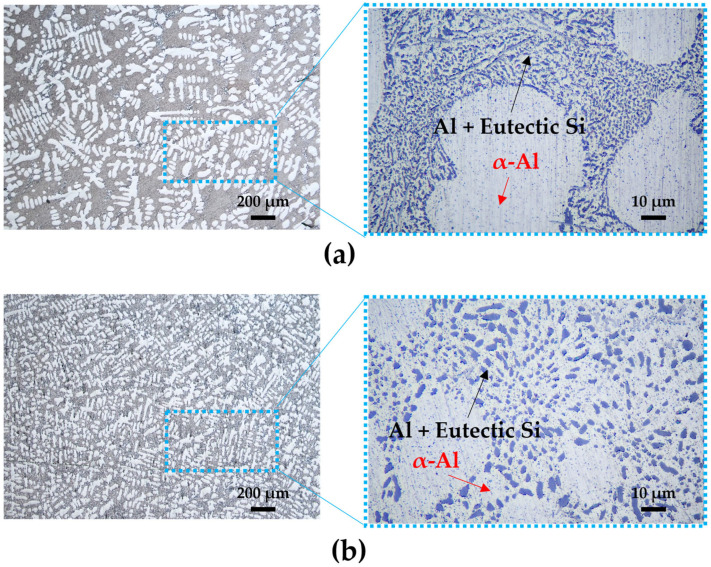
Microstructure of A356 alloy before and after solution treatment: (**a**) A356, (**b**) A356-S.

The microstructural changes in the A356 alloy before and after solution treatment are shown in Figure 2. In the microstructure before solution treatment, silicon primarily exists as eutectic silicon in an acicular form along with α-Al in a dendritic structure. This microstructure leads to stress concentration within the material, adversely affecting mechanical properties such as ductility, impact resistance, and fatigue life, thereby limiting its industrial applications [19,23]. After solution treatment, it was observed that the shape of the silicon particles became spheroidized. The spherical Si in this eutectic microstructure has been reported to improve mechanical properties such as ductility [24,25]. It also homogenizes the distribution of alloy elements in the α-Al matrix by dissolving and forming solid solutions of intermetallic compounds formed during solidification [25,26,27,28]. This can promote Mg_2_Si precipitation during aging treatment, which can improve the mechanical properties of the A356 alloy after aging treatment. Therefore, the cast A356 alloy requires solution treatment at 525 °C.

**Figure 3:**
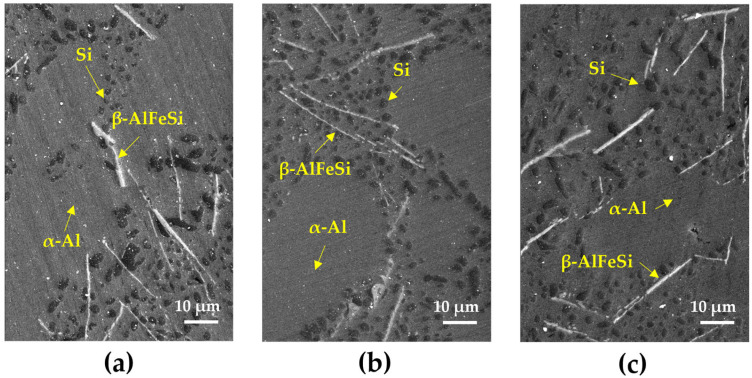
Microstructure of A356 alloy according to aging treatment time: (**a**) A356-SA3; (**b**) A356-SA6; (**c**) A356-SA9.

The microstructural changes resulting from the aging treatment were observed using scanning electron microscopy (SEM), as shown in Figure 3. It was confirmed that the eutectic silicon particles continued to retain their spheroidized form, even after aging treatment. The spheroidizing of silicone in the eutectic structure occurred via atomic diffusion and rearrangement to stabilize the internal structure. Moreover, the spherical silicon particles achieved a more thermodynamically stable state due to the reduction in surface energy, which also influences the mechanical properties [29]. After aging treatment, β-AlFeSi precipitation in needle form can be found in the microstructure. This phase has been reported to exhibit more cathodic behavior compared to the α-Al matrix, leading to localized corrosion at the interface [30,31]. However, this β-AlFeSi precipitation did not increase with the aging duration due to its limited Fe content (in the alloy). Moreover, the precipitation of the Mg_2_Si phase (which is the major strengthening mechanism of magnesium-containing Al-Si alloy) cannot be found in the SEM image.

**Figure 4:**
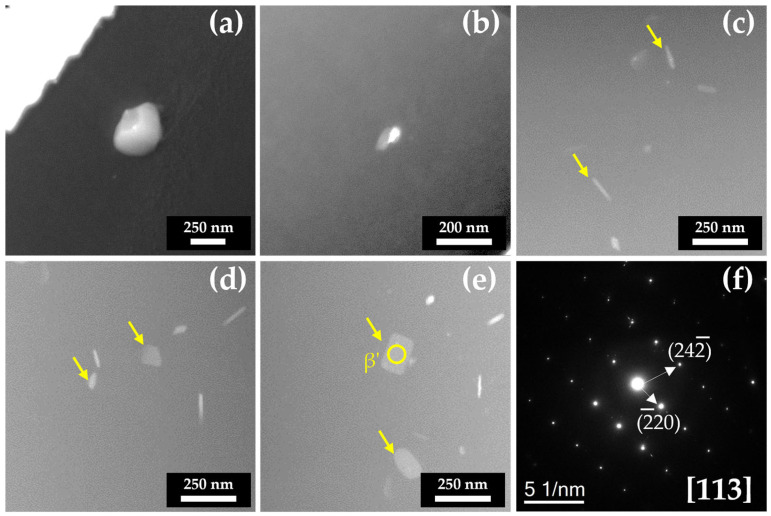
TEM images of A356 alloy samples and SAD pattern of Mg_2_Si precipitate: (**a**) A356; (**b**) A356-S; (**c**) A356-SA3; (**d**) A356-SA6; (**e**) A356-SA9; (**f**) SAD pattern obtained from yellow circle in (**e**).

To verify the presences of the Mg_2_Si precipitate, transmission electron microscopy (TEM) was conducted. Figure 4 presents the annular dark-field scanning TEM (ADF-STEM) images of as-cast and heat-treated A356 alloy and the selected area diffraction (SAD) pattern of disc-shaped precipitates. Mg_2_Si particles of about 397 nm were observed in the as-cast specimen (Figure 4a). In Figure 4b, it can be seen that smaller Mg_2_Si particles were detected after solution treatment. The diameter of the particle was 68 nm, which was a 5.8-times reduction compared to the as-cast specimen. It indicated that Mg_2_Si particles were dissolved and diffused in the α-Al matrix during solution treatment. Even though the Mg_2_Si phase can be dissolved over a temperature of 467 °C (Figure 1a), it still remained in the matrix after the solution treatment at 525 °C. The equilibrium phase composition by JmatPro (Figure 1a) does not consider the time and cooling to room temperature. This indicates that 4 h at 525 °C is not sufficient to totally dissolve the Mg_2_Si particles formed during casting. In addition, during the cooling of solid-treated sample, the Mg_2_Si phase can be precipitated, since it can be formed under a temperature of 467 °C (Figure 1a). After aging treatment, rod or disc-like precipitates were observed (Figure 4c–e). Mg_2_Si precipitates were observed after 3 h aging treatment at 190 °C, indicating that this temperature can promote precipitation (Figure 1b). As the holding time increased, disc-like precipitates were observed in 3 and 6 h treated specimens. The SAD pattern analyzed from the yellow circle in Figure 4e is presented in Figure 4f. Diffraction analysis results indicate that the disc-like precipitate is β’-Mg_2_Si, which is consistent with the results reported in a previous study [32]. The TEM analysis results confirmed the presence of Mg_2_Si particles in the A356 alloy. However, the TEM analysis does not provide any information about how many Mg_2_Si particles were precipitated.

### 3.2. Precipitates and Hardness

**Figure 5:**
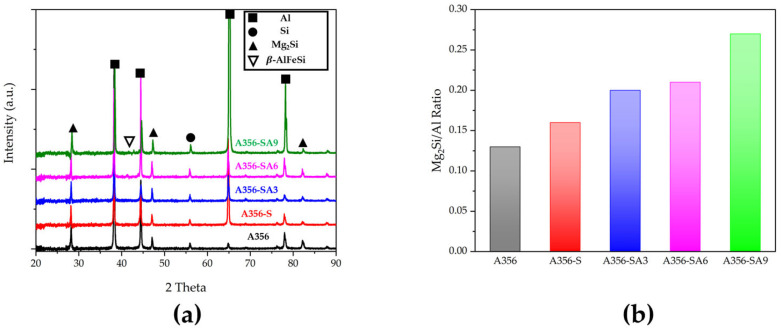
XRD Analysis of A356 alloy (**a**) XRD spectrum, (**b**) Mg_2_Si (111) and Al (111) XRD peak ratios.

Figure 5a shows the XRD pattern of A356 alloy with solution treatment following aging treatment. The XRD diffraction analysis results indicate that Al and Si are the primary phases present in all specimens, with peaks corresponding to Mg_2_Si precipitates clearly identified. To quantitatively analyze the precipitation of Mg_2_Si, the intensity ratio of Mg_2_Si (111) and Al (111) was calculated, and the results are shown in Figure 5b. The Mg_2_Si peak can be found in the as-cast sample, indicating that the precipitation of Mg_2_Si can occur during the solidification of A356 alloy. After the solution treatment, the intensity ratio increased from 0.13 to 0.16, indicating that more Mg_2_Si is precipitated in the microstructure. The intensity ratio was further increased by the aging treatment, and then the intensity ratio reached its maximum value (0.27) after the longest aging period of 9 h. It has been reported that the amount of Mg_2_Si precipitate increases with aging temperature and time [32,33]. The rise in the peak intensity ratio indicates that our peak analysis is consistent with previous studies. Furthermore, the precipitation during solution treatment and aging is expected to accelerate the precipitation hardening of the alloy [34]. In the case of the β-AlFeSi phase, it was observed through the SEM image, but no clear diffraction peak was detected in the XRD analysis. This is due to the small fraction and low diffraction intensity of the β-AlFeSi phase not being detected (Figure 1a).

The XRD analysis results confirmed that the heat treatment conditions significantly influenced the microstructure of the A356 alloy by promoting the formation of Mg_2_Si precipitates. In order to confirm how these microstructural changes affected the mechanical properties of the material, the hardness measurement was conducted on each specimen.

**Figure 6:**
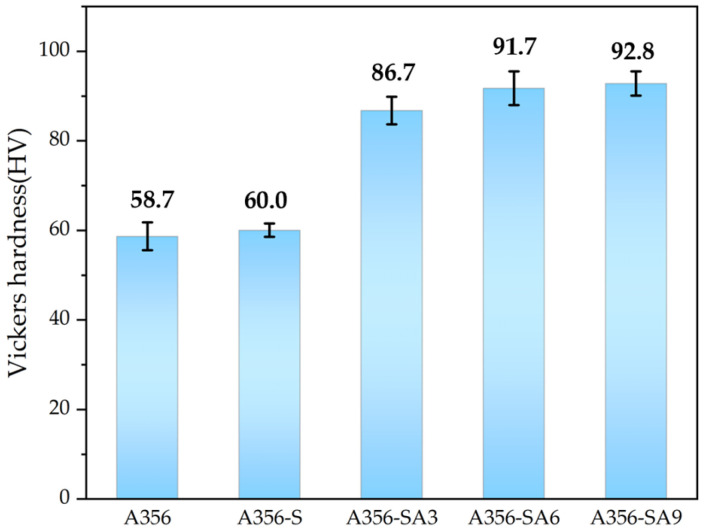
Vickers’ hardness (H_V_) measurements of A356 alloy under different heat treatment conditions.

The polishing with sandpaper (grit #2000) was conducted to measure the hardness, and the hardness of the polished specimen was measured using a Vickers’ hardness tester. The test conditions involved applying an indentation load of 150 g·f for 15 s. The average values of five measurements for each specimen are presented in Figure 6. The hardness of the as-cast specimen was recorded at 58.6 Hv.

After solution treatment, Mg and Si formed a supersaturated solid solution, in which they were uniformly dissolved in the aluminum base material, and this state was maintained through rapid cooling. Moreover, during the solution treatment, the internal stress induced by irregular solidification is relieved, which contributes to the decrease in hardness. However, more Mg_2_Si precipitated during the solution treatment, which contributed to the increase in hardness. Therefore, the hardness of A356 with solution treatment shows a similar value of 60.0 HV. With aging, the precipitation of Mg_2_Si is accelerated so that the hardness of A356 increases with the aging duration, such as 86.7 HV, 91.7 HV and 92.8 HV for aging durations of 3, 6 and 9 h (Figure 6). This increase in hardness with aging treatment time shows similar behavior to that reported previously [35,36,37].

In this study, XRD analysis was utilized to investigate the microstructure and phase distribution changes in the A356 alloy containing Mg. Based on the XRD analysis results and the ratio of the precipitated phase and the primary phase, it was confirmed that the solution treatment effectively dissolved supersaturated Mg and Si to form a uniform solid solution and that fine precipitates, such as Mg_2_Si, were formed and grew over time during the aging process. It was observed that mechanical hardness gradually improved as the Mg_2_Si precipitates grew and stabilized as the aging treatment and time increased. It can be confirmed that this heat treatment mechanism and the behavior of the precipitated phase have a significant effect on improving the enhancement of the material [32].

### 3.3. Corrosion Resistance

**Figure 7:**
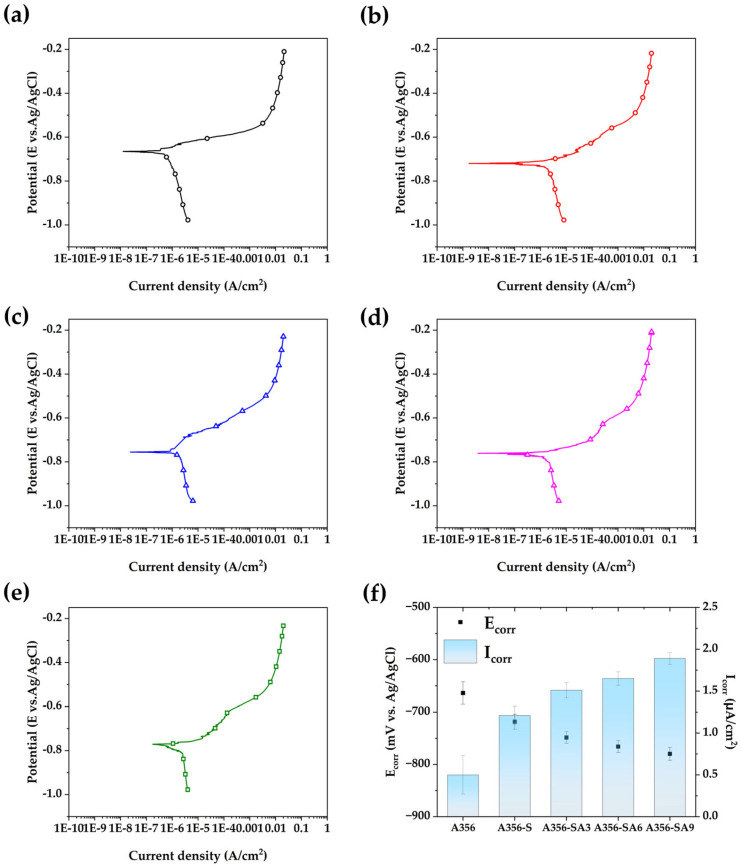
Potentiodynamic curves of specimens under different heat treatment conditions: (**a**) A356; (**b**) A356-S; (**c**) A356-SA3; (**d**) A356-SA6; (**e**) A356-SA9; (**f**) polarization values.

The potentiodynamic polarization curves of the A356 sample in 3.5 wt.% NaCl solution are shown in Figure 7. In addition, the corrosion current density (I_corr_) and corrosion potential (E_corr_) were estimated by Tafel fitting of potentiodynamic polarization curves, and they are summarized in Table 3. The corrosion potential and current density of as-casted A356 alloy are −663.81 mV and 0.5 μA/cm^2^, respectively. After the solution treatment, the corrosion potential decreased to −718.27 mV and the corrosion current density increased to 1.21 μA/cm^2^, indicating the deterioration of corrosion resistance. A further decrease in the corrosion potential was shown with aging treatment, such as −748.52 mV, −765.91 mV and −779.82 mV for 3, 6, and 9 h, respectively. Moreover, the corrosion current density increased to 1.512 μA/cm^2^, 1.65 μA/cm^2^, and 1.89 μA/cm^2^ for aging durations of 3, 6 and 0 h, respectively. The decrease in corrosion potential indicates the metal surface working as an anode for corrosion changed to be more active. Moreover, the increase in corrosion current density means that the corrosion rate increases with solution treatment and aging. These results also indicate the aging treatment causes negative effects on corrosion resistance, while the hardness can be increased. Generally, the solution treatment is well known to form a uniform solid solution of alloy, but the solution treatment for A356 could not totally remove the eutectic phase as well as Mg_2_Si precipitation. Moreover, the precipitation of Mg_2_Si is strengthened by the solution treatment, which also occurred with aging treatment. Therefore, the decrease in corrosion resistance as a result of the solution treatment and aging was attributed to the precipitation of the Mg_2_Si phase.

**Figure 8:**
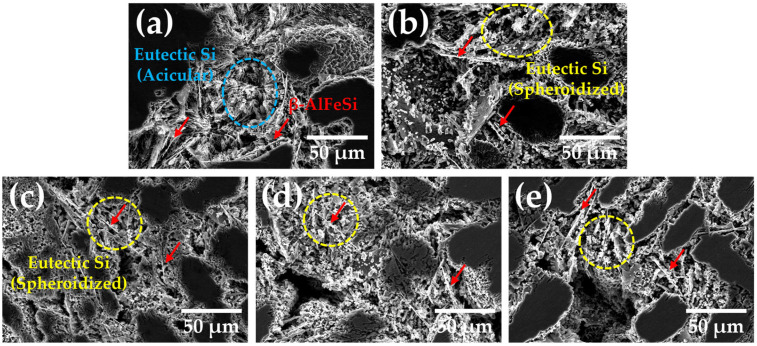
The SEM images of corroded surfaces after potentiodynamic polarization: (**a**) A356; (**b**) A356-S; (**c**) A356-SA3; (**d**) A356-SA6; (**e**) A356-SA9. Dotted circle and red arrow indicate the corroded area and β-AlFeSi phase.

SEM images of the corroded surfaces of all specimens after potentiodynamic polarization testing are shown in Figure 8. In the as-cast specimen in Figure 8a, acicular-like eutectic Si and undissolved β-AlFeSi phases were observed. After solution treatment, spheroidal eutectic Si and β-AlFeSi phases remained, indicating that these phases are noble than α-Al matrix, and the Galvanic corrosion occurred. The electrochemical potential difference between eutectic Si, β-AlFeSi and α-Al matrix causes Galvanic corrosion at the interface [38,39]. In addition, the β-AlFeSi phase has a relatively low volume fraction (less than 1%), which is almost constant over temperature. Therefore, the β-AlFeSi phase does not significantly affect the corrosion property of A356 alloy. In Figure 8c–e, eutectic Si and β-AlFeSi phases were still observed, even after aging treatment, regardless of aging treatment time. The dissolution of the α-Al matrix was concentrated on the interface between the α-Al matrix and eutectic Si or β-AlFeSi, regardless of the heat treatment conditions. All specimens used in this study had needle-like β-AlFeSi and eutectic Si, which remained after solidification. The corrosion mechanism of A356 involves localized corrosion through the initial solidification microstructure, independent of Mg_2_Si precipitation.

**Figure 9:**
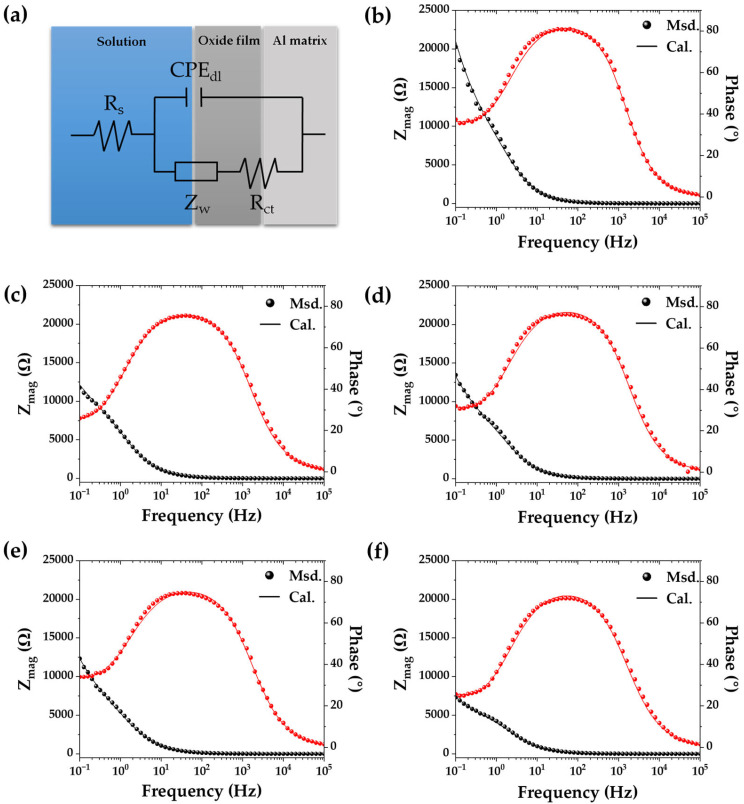
EIS results of A356 alloy in KCl solution: (**a**) equivalent circuit; (**b**) A356; (**c**) A356-S; (**d**) A356-SA3; (**e**) A356-SA6; (**f**) A356-SA9.

Electrochemical impedance spectroscopy measurements of A356 alloy in corrosion NaCl solution were carried out for detailed corrosion resistance analysis. The equivalent circuit and Bode plots with measured and simulated data are shown in Figure 9. The estimated results using model fitting are also summarized in Table 4. The equivalent circuit used for EIS analysis was composed of solution resistance (R_s_), charge transfer resistance (R_ct_), constant phase element at electrochemical double layer (CPE_dl_), and Warburg impedance (Z_w_). The CPE_dl_, which is used to describe unideal capacitor behavior, is composed of capacitance (C_dl_) and exponent (n). The charge transfer resistance corresponds to the resistance to corrosion with an ionization of aluminum from the alloy surface. The R_s_ corresponds to the solution resistance for 3.5 wt.% NaCl solution; thus, any significant difference in each test was not recognized. The heat treatment of A356 alloy resulted in significant changes in R_ct_ and C_dl_. The R_ct_ of as-casted A356 alloy was 8558 Ω∙cm^2^, and then decreased to 7941 Ω∙cm^2^ after the solution treatment. Then, the R_ct_ was further decreased to 7063, 6604 and 4647 Ω∙cm^2^ with an increase in the aging duration for 3, 6, and 9 h, respectively. The decrease in R_ct_ is attributed to the formation of Mg_2_Si. Mg is usually used as a substitutional alloying element in aluminum to improve strength and corrosion resistance. Less than ~1 wt.% of Mg is known to be effective in corrosion resistance, work hardening, weldability and strength. However, the precipitation of Mg_2_Si decreases the substitutional contents of Mg in the aluminum matrix. Therefore, the corrosion resistance decreases as the magnesium, an element that enhances corrosion resistance, becomes depleted in the aluminum matrix due to the precipitation of Mg_2_Si [31,40]. The C_dl_, indicating the capacitance element at the interface between the reactive metal surface and electrolyte, is increased by the solution treatment and following aging treatment. This increase in capacitance is also due to the increase in the active metal area forming the electrochemical double layer through the depletion of Mg in the aluminum matrix during the heat treatments. The decrease in R_ct_ and increase in C_dl_ indicate the corrosion resistance of the A356 alloy is decreased by the heat treatments forming the Mg_2_Si phase, and they are in good agreement with potentiodynamic polarization tests (Figure 7).

## 4. Summary

In this study, the microstructural changes, mechanical properties, and corrosion resistance of manufactured specimens were analyzed by conducting solution and aging treatments on A356 aluminum alloys.

In the A356 alloy that underwent solution treatment and aging, it was confirmed that the Si particles transformed from a needle-like structure to a more spherical shape. This change in microstructure can enhance mechanical properties by reducing the stress concentration. Additionally, the XRD analysis results confirmed that the precipitation phase Mg_2_Si was formed during the aging process, leading to an effective precipitation hardening effect.

As a result of hardness measurement, the hardness of A356 was 58.6 HV, and the hardness of A356-SA9 was 92.38 HV, which increased by approximately 57.6%. It was confirmed that finely distributed Mg_2_Si precipitates were formed, and mechanical properties were improved, depending on the aging duration.

As a result of evaluating corrosion resistance through electrochemical analysis, it was confirmed that the corrosion resistance was reduced due to the formation of the Mg_2_Si phase, depleting substitutional alloying Mg atoms in the aluminum matrix.

In conclusion, solution and aging treatments, leading to precipitation of the Mg_2_Si phase, effectively improve the mechanical properties of casted A356 alloy such as hardness, but they contribute to the decrease in corrosion resistance. Therefore, the design of heat treatment for A356 alloys for mechanical properties should consider the risk of corrosion resistance. Therefore, optimizing the precipitation of phases formed during heat treatment is essential to achieving a balance between corrosion resistance and mechanical properties. In particular, by refining the heat treatment process of A356 alloys, this study demonstrates that A356 can serve as a key candidate material, with both excellent mechanical properties and corrosion resistance in various industries where lightweight structural materials are required.

## Figures and Tables

**Table 1:** Chemical compositions of A356 alloy.

Element	Si	Cu	Mg	Fe	Ti	Mn	Zn	Bal
(wt.%)	7.00	0.25	0.45	0.6	0.25	0.35	0.35	Al

**Table 2:** Experimental parameters of heat treatment for A356 alloy.

Sample	Alloy	Solution Treatment Temperature (°C)	Time (h)	Aging Treatment Temperature (°C)	Time (h)
A356	A356	-	-	-	-
A356-S		525	4	-	-
A356-SA3		525	4	190	3
A356-SA6		525	4	190	6
A356-SA9		525	4	190	9

**Table 3:** Measured potential and current density values after potentiodynamic polarization tests.

	A356	A356-S	A356-SA3	A356-SA6	A356-SA9
E_corr_ (mV)	−663.81 ± 21.48	−718.27 ± 14.31	−748.52 ± 10.98	−765.91 ± 11.22	−779.82 ± 12.34
I_corr_ (μA/cm^2^)	0.50 ± 0.23	1.21 ± 0.11	1.51 ± 0.09	1.65 ± 0.08	1.89 ± 0.07

**Table 4:** EIS analysis results according to heat treatment conditions.

Sample	R_s_ (Ω·cm^2^)	R_ct_ (Ω·cm^2^)	Q_dl_ (μ/cm^2^)	n_ct_
A356	12.95 ± 0.19	8558 ± 44.81	10.62 ± 0.11	0.86 ± 0.01
A356-S	12.26 ± 0.43	7941 ± 70.15	15.73 ± 0.20	0.92 ± 0.01
A356-SA3	12.42 ± 0.28	7063 ± 31.07	16.20 ± 0.06	0.91 ± 0.01
A356-SA6	12.19 ± 0.45	6604 ± 28.96	22.44 ± 0.30	0.88 ± 0.02
A356-SA9	12.80 ± 0.32	4647 ± 42.50	24.26 ± 0.42	0.88 ± 0.03

## Data Availability

The original contributions presented in the study are included in the article, further inquiries can be directed to the corresponding authors.
